# Bridging Machine Learning and Clinical Endpoints: A METABRIC-Informed Simulation Study of Missing Data Imputation for RECIST-Based Best Overall Response

**DOI:** 10.3390/diagnostics16121853

**Published:** 2026-06-15

**Authors:** Fangya Tan, Bowen Long

**Affiliations:** School of Analytics & Computational Sciences, Harrisburg University of Science and Technology, Harrisburg, PA 17101, USA; ftan1@my.harrisburgu.edu

**Keywords:** LSTM, machine learning, MissForest, METABRIC, oncology, RECIST, BOR, missing data, MAR, MNAR, non-responder imputation

## Abstract

**Background**: Missing data, particularly progression-driven dropout, introduces substantial bias in longitudinal oncology studies, directly impacting response classification based on RECIST criteria. While machine learning-based imputation methods are increasingly used, their performance is rarely evaluated in a clinically interpretable framework centered on patient-level endpoints such as Best Overall Response (BOR). **Methods**: We propose a clinically grounded evaluation framework based on RECIST 1.1 focused on patient-level Best Overall Response classification. Longitudinal tumor trajectories were simulated for 270 patients (1:1, HER2+ and HER2−) across nine follow-up visits using both Gompertz and Stein–Fojo growth models, resulting in 2700 patient-visit observations. Realistic missingness was introduced through a combination of random mechanisms and progression-driven dropout. Three machine learning imputation models, long short-term memory (LSTM), MissForest, and Multiple Imputation (MI) were evaluated under both direct (MAR-based) and non-responder imputation strategies. Performance was assessed using BOR classification metrics, including accuracy and Cohen’s kappa. **Result**: Across both simulation frameworks, imputation substantially improved BOR classification performance. Under the Gompertz model, accuracy increased from 0.84–0.89 with direct imputation to 0.94–0.99 with non-responder imputation, with corresponding kappa improvements from 0.73–0.82 to 0.90–0.99. Similar trends were observed under the Stein–Fojo model (accuracy: 0.82–0.84 vs. 0.91–0.96; kappa: 0.69–0.72 vs. 0.86–0.94). Across all evaluated methods, NRI improved classification performance by approximately 10 percentage points in accuracy and up to 17 percentage points in kappa. The improvement was observed consistently across both tumor growth models and different missingness scenarios, demonstrating the robustness of the findings. **Conclusions**: This study demonstrates that successful handling of missing data depends not only on the imputation method itself, but also on the choice of a clinically meaningful endpoint and appropriate estimand strategies aligned with the underlying missing data assumptions. In the METABRIC-derived simulations, clinically informed handling of progression-related missingness substantially improved RECIST-based BOR classification across all evaluated methods, suggesting that appropriate endpoint selection and the corresponding estimand strategy for missing data handling may have a greater influence on classification performance than the choice among the imputation models applied.

## 1. Introduction

In oncology clinical trials, overall survival (OS) and progression-free survival (PFS) are considered the gold-standard endpoints for evaluating treatment benefit [1]. Although these are time-to-event outcomes, their evaluation is closely linked to tumor response assessed during study visits. In particular, the Response Evaluation Criteria in Solid Tumors (RECIST 1.1) provides a standardized framework that translates longitudinal tumor size measurements into clinically interpretable categories, including complete response (CR), partial response (PR), stable disease (SD), and progressive disease (PD) [2].

Because the RECIST response categories are derived from measurements collected repeatedly over time, longitudinal tumor dynamics provide the critical link between patient-level disease evolution and clinical outcomes. Research has shown that longitudinal tumor sizes changes are strongly associated with OS and PFS [3,4].

However, in clinical trials, longitudinal tumor assessments are often analyzed under a complete case framework, whereas in real-world settings these measurements are frequently incomplete. Under the Missing Not At Random (MNAR) mechanism, patients with more aggressive disease are more likely to discontinue follow-up upon reaching a progression event rather than random missing. Hence, if not properly addressed, such missing data can bias patient-level response classification, ultimately affecting the interpretation of survival outcomes, like OS and PFS [5,6].

Therefore, accurately characterizing tumor size trajectories is essential for reliable clinical evaluation, as these trajectories capture the dynamic evolution of treatment response over time. Compared with static summaries at a specific time point, modeling tumor size dynamics provides a more comprehensive and clinically meaningful representation of disease progression. However, this process is often complicated by missing data, which can disrupt the continuity of tumor trajectories and compromise individual response assessments.

To address this challenge, robust methods are needed to handle missing data while preserving the underlying non-linear tumor dynamics and clinically interpretable endpoints. In this study, we evaluate whether advanced machine learning-based imputation methods can improve patient-level BOR assessment from incomplete longitudinal tumor measurements under clinically realistic, progression-driven missingness scenarios.

This motivation is supported by clinical evidence that underscores the strong relationship between tumor size and survival outcomes. Tumor burden serves not only as a marker of disease progression but also as a key prognostic factor in oncology. In a recent study [3], examined the prognostic value of baseline tumor size (BTS) in 583 patients with advanced melanoma treated with pembrolizumab in the KEYNOTE-001 trial. Patients with BTS below the median experienced substantially higher objective response rates (44% vs. 23%) and significantly longer overall survival (HR 0.38; *p* < 0.001). Crucially, in multivariate analyses adjusting for established prognostic factors including LDH level, ECOG performance status, and disease stage, BTS remained an independent predictor of OS, demonstrating that tumor size carries prognostic information beyond what is captured by standard clinical covariates. These findings demonstrate that tumor size carries prognostic information beyond standard clinical covariates and is closely linked to survival outcomes in oncology.

In breast cancer specifically, tumor size has long been recognized as one of the strongest predictors of patient survival. In particular, reference [4] demonstrated, across three independent breast cancer populations totaling thousands of patients, that survival is a direct and quantifiable function of tumor size at diagnosis, independent of detection method. Their analysis further showed that reductions in tumor size over time were associated with an approximately 35% absolute improvement in survival over five decades. Taken together, these findings reinforce the fundamental relationship between tumor burden and survival outcomes. They also highlight that tumor size is not only a static prognostic factor at diagnosis but a clinically meaningful variable that evolves over time. As such, longitudinal tumor size trajectories provide a central and outcome-relevant measure for understanding treatment response and disease progression in oncology trials.

Beyond treating tumor size as a continuous variable, recent studies have focused on clinically interpretable representations of tumor dynamics. In particular, Best Overall Response (BOR), derived from longitudinal tumor measurements using RECIST criteria, provides a widely used categorical summary of treatment response in oncology trials. For example, a recent study analyzed individual patient data from 13 oncology trials and showed that a trichotomized tumor response classification (complete/partial response, stable disease, and progressive disease) predicted overall survival as effectively as alternative response categorizations [7]. Their findings support the clinical relevance and interpretability of multiple category BOR classifications, which capture meaningful distinctions in tumor dynamics that may be obscured by binary response definitions. However, the analysis relied on complete-case methods, restricting evaluation to patients with available tumor assessments at predefined landmark time points and without explicitly addressing missing data mechanisms. Because BOR is derived from longitudinal tumor trajectories, missing assessments, particularly those associated with disease progression or treatment discontinuation, may substantially affect response categorization and downstream clinical interpretation.

Moreover, reference [8] examined the relationship between RECIST-based tumor size changes and overall survival across 570 patients enrolled in 24 phase I oncology trials. Their findings revealed a near-linear, continuous relationship between percent change in tumor burden and survival, with no identifiable inflection points at the RECIST thresholds for partial response (−30%) or progressive disease (+20%). These results indicate that CR/PR/SD/PD categories represent a clinically convenient discretization of an inherently continuous tumor–survival relationship, rather than reflecting biologically distinct transitions at those specific thresholds. In this context, Best Overall Response (BOR) is best understood as a clinically interpretable summary derived from the best percentage change from baseline, rather than an independently assigned endpoint capturing a qualitatively different disease state. Preserving the integrity of the underlying tumor size measurements from which BOR is derived is therefore essential when evaluating treatment response. However, like the prior studies discussed above, reference [8] relied on complete-case methods, excluding patients with missing tumor assessments without adjustment for the missingness mechanism, and characterized response using a single summary metric rather than the full longitudinal trajectory of tumor dynamics.

To further explore tumor dynamics and their link to clinical outcomes, model-based approaches have been developed. A notable example is [9], who characterized longitudinal tumor size trajectories and predicted overall survival in metastatic colorectal cancer using a tumor growth inhibition (TGI) framework combining exponential decay and linear growth components. The study translated phase II tumor response data into phase III survival predictions using a parametric survival model linking change in tumor size at week 7 to overall survival, with a median of 431 days predicted versus 401 days observed for the capecitabine arm. Importantly, this work focused on population-level survival outcomes: while tumor trajectories were modeled as an internal mechanism, the primary reported results were aggregated survival distributions across treatment arms rather than individual patient-level response classifications. Furthermore, missing data mechanisms were not explicitly addressed despite their relevance in longitudinal oncology studies; the analysis was conducted on available complete data without characterization of the dropout process. Thus, although reference [9] highlights the value of longitudinal tumor dynamics as a surrogate for survival, they do not assess how incomplete trajectories arising from missed assessments, early discontinuation, or progression-related dropout might distort either the tumor growth model or the downstream survival estimates.

Reference [10] is particularly relevant to the present study, as it linked longitudinal tumor size trajectories to overall survival (OS) and progression-free survival (PFS) in 1985 patients from six randomized breast cancer trials. Tumor burden, defined as the sum of the longest diameters of target lesions, was modeled using a Gompertz growth and exponential decay framework, similar to the approach adopted in this study. Changes in tumor size over time were significant predictors of both OS and PFS, with greater tumor shrinkage consistently associated with improved outcomes. These findings demonstrate that longitudinal tumor dynamics provide clinically meaningful information for evaluating treatment response. However, missing data remained an important limitation. Key variables, including HER2 and PR status, could not be included in some analyses because of substantial missingness. More broadly, the study did not evaluate missing data mechanisms, compare imputation strategies, or assess how incomplete tumor trajectories influence RECIST-based Best Overall Response (BOR) classification. These gaps motivate the present study, which focuses on the impact of missing data handling on patient-level response assessment.

Taken together, existing studies have established the clinical importance of the tumor size–survival relationship but have largely assumed complete longitudinal tumor data, leaving unanswered how different missing data mechanisms and imputation strategies influence patient-level BOR classification over time.

To illustrate the potential impact of missing data on clinical conclusions, recent empirical studies have demonstrated that missingness is not only prevalent but also clinically meaningful. Reference [6] analyzed over 4.4 million patients with the three most common cancers in the United States, non-small cell lung cancer (NSCLC), breast cancer, and prostate cancer, using the National Cancer Database and found that missing data were associated with significantly worse overall survival across all three groups. In NSCLC, patients with missing data had a 2-year overall survival of 33.2% compared to 51.6% for those with complete data, an absolute difference of 18.4 percentage points. In breast cancer, which is directly relevant to the present study, 54.7% of patients had missing data, and 2-year overall survival was 93.2% in those with missing data versus 93.9% in those with complete data, a difference that was also statistically significant (*p* < 0.001).

This substantial survival gap suggests that missingness is likely informative and may reflect underlying patient dropout, disease progression, and incomplete follow-up, rather than occurring at random. Consequently, ignoring or inadequately handling missing data may lead to biased estimation of treatment effects and misleading clinical conclusions. These findings highlight a recurring challenge in oncology studies and underscore the need for robust and clinically appropriate methods to handle missing data, particularly in the context of longitudinal tumor assessments.

Recent studies have explored the use of machine learning for missing data imputation in clinical datasets. For example, reference [11] provided one of the earliest systematic comparisons of statistical and machine learning imputation methods using the El Álamo-I breast cancer dataset (N = 3679). Conventional statistical approaches, including mean imputation, hot-deck, and Multiple Imputation, were evaluated alongside machine learning methods such as multi-layer perceptron (MLP), self-organizing maps (SOM), and k-nearest neighbors (KNN), with listwise deletion serving as a baseline comparator.

Imputation performance was assessed through downstream prediction of early cancer relapse using neural network classifiers. The results showed significant differences across methods (Friedman test *p* = 0.0091), with MLP, KNN, and SOM achieving significantly higher AUC compared to listwise deletion (*p* < 0.01), while conventional statistical methods did not demonstrate comparable improvements. These findings suggest that machine learning-based imputation can improve predictive performance for binary relapse outcomes in breast cancer datasets where missing data would otherwise reduce model accuracy. However, the missingness was treated as naturally occurring and was not explicitly characterized. As a result, it remains unclear whether these methods perform similarly under defined missingness mechanisms or whether they can reliably recover clinically meaningful oncology endpoints.

Recent work has examined the role of imputation within predictive modeling pipelines, focusing not only on method selection but also on when imputation should be applied. Reference [12] evaluated the ordering of imputation, feature selection, and classification across three healthcare datasets, including breast cancer, heart disease, and diabetes, using seven imputation methods under missingness levels of 10%, 15%, 20%, and 25% introduced under a missing completely at random framework.

Across the three datasets, MissForest consistently achieved the lowest imputation error and improved downstream classification performance when applied prior to feature selection and model training, as measured by recall, precision, F1 score, and accuracy. This suggests that preserving the full set of variables during imputation enhances data reconstruction and predictive performance. However, missingness was restricted to missing completely at random, without examining more realistic mechanisms such as missing not at random.

To further evaluate random forest-based imputation under complex data conditions, reference [13] compared MissForest and CALIBERrfimpute in datasets with non-normal distributions, nonlinear relationships, and variable interactions. Although both methods achieved high predictive accuracy, MissForest produced biased regression estimates and reduced confidence interval coverage, particularly for skewed variables. CALIBERrfimpute generally showed better performance for coefficient estimation but still exhibited bias in some scenarios. Similar findings were observed in a real-world oncology application, where MissForest demonstrated high concordance in predicting tumor diameter and serum AFP values. However, accurate reconstruction of missing values did not necessarily translate into unbiased downstream inference. Together, these results suggest that imputation methods should be evaluated not only by prediction accuracy, but also by their impact on clinically meaningful endpoints. This limitation motivates the need to assess imputation strategies under realistic missingness mechanisms and endpoint-focused evaluation frameworks.

In longitudinal clinical data, alternative approaches have been proposed to directly model missingness. For example, a recent study addressed this by developing GRU-D, a recurrent neural network that directly incorporates missingness into the model [14]. It uses a masking vector to indicate observed values and a time interval vector to capture how long it has been since the last measurement. Rather than imputing values separately, GRU-D treats both the presence and timing of missingness as predictive signals and outperformed approaches that first imputed data using methods such as MissForest or MICE. This idea is directly relevant to oncology. In breast cancer trials, early dropout is often driven by disease progression, meaning missing tumor measurements are informative rather than random. Imputation methods that ignore this pattern may produce overly optimistic trajectories and bias both RECIST-based response classification and treatment effect estimates. Although GRU-D was developed for prediction rather than trajectory recovery, it highlights that missingness should be modeled rather than ignored, motivating the use of LSTM-based approaches in this study. Methods that leveraged temporal information generally outperformed traditional statistical approaches, particularly for longer time series. 

Another line of work has examined deep learning methods for imputing multivariate time series data [15]. Benchmarked eight deep learning imputation methods across five healthcare datasets, addressing limitations in earlier studies that focused on a small number of datasets and primarily missing completely at random settings. Plain LSTM was used as the standard deep learning baseline, against which more advanced architectures, including BRITS, CATSI, and bidirectional LSTM variants, were compared. These findings are relevant to the present study because they support the use of LSTM as a well-established method for imputing longitudinal data. Tumor measurements are inherently sequential, and accurate reconstruction of longitudinal tumor trajectories is important for downstream RECIST-based Best Overall Response (BOR) classification.

More recently, another study conducted a systematic review of 46 studies examining missing data handling methods in electronic health records [16]. The review compared statistical and machine learning approaches across different dataset types, missingness mechanisms, and evaluation metrics. Across 26 studies with direct comparisons, machine learning and deep learning methods consistently outperformed traditional statistical imputation, with the advantage most pronounced in longitudinal data where temporal dependencies play a key role. The review also showed that imputation performance depends strongly on data structure, with methods effective for cross-sectional data performing poorly on longitudinal data. These findings directly support the design of the present study. Tumor size measurements in breast cancer trials are inherently longitudinal, with serially dependent observations within each patient. This places the problem in a setting where machine learning methods, particularly sequence-based models such as LSTM, are expected to perform well. At the same time, the review highlights an important gap. Despite strong evidence favoring machine learning imputation in longitudinal data, no prior study has evaluated whether these methods preserve clinically interpretable oncology endpoints.

Across prior studies, missing data remains a persistent challenge in oncology, where longitudinal tumor assessments are frequently incomplete due to dropout, disease progression, and irregular follow-up. Because these missing data are often informative, inappropriate handling can lead to biased estimates of treatment response and compromise clinical interpretation.

In oncology trials, treatment response is commonly summarized using RECIST-based Best Overall Response (BOR), a patient-level endpoint that plays an important role in both clinical and regulatory decision-making. Despite its importance, most studies evaluating missing data methods focus on reconstruction accuracy or predictive performance rather than their impact on BOR classification. As a result, the clinical relevance of many imputation approaches remains unclear.

Machine learning methods such as LSTM and MissForest offer flexible approaches for imputing incomplete longitudinal data and have demonstrated promising predictive performance in a variety of settings. However, limited work has evaluated these methods using clinically meaningful oncology endpoints. Furthermore, most studies do not explicitly consider clinically realistic missingness mechanisms, particularly progression-related missingness, which is common in oncology trials and often resembles a missing-not-at-random (MNAR) process.

To address these limitations, we developed a clinically grounded simulation framework that integrates longitudinal tumor growth models (Gompertz and Stein–Fojo), RECIST-based BOR assessment, and estimand-aware missing data handling strategies. Within this framework, three imputation methods, LSTM, MissForest, and Multiple Imputation (MI), were evaluated under both direct imputation and non-responder imputation (NRI). Performance was assessed using BOR classification metrics, including accuracy and Cohen’s kappa.

This framework enables systematic evaluation of how endpoint definitions and missing data assumptions, together with their corresponding handling strategies, influence treatment response classification, representing a key methodological contribution of this study.

## 2. Materials and Methods

### 2.1. Data Preprocessing

This study simulates longitudinal tumor size trajectories to evaluate missing data handling methods under clinically realistic trial conditions. The simulation framework is anchored to the publicly available METABRIC dataset, which includes 2509 breast cancer patients with rich clinical and biological variables [17]. Observed baseline characteristics were used to initialize patient-level tumor profiles, ensuring that the simulated data reflect realistic clinical heterogeneity.

To construct a cohort resembling a controlled clinical trial population with complete baseline information, patients with missing values in key variables required for simulation and matching (including tumor size, age, ER status, HER2 status, etc.) were excluded. After this filtering step, 135 HER2-positive patients remained available. Because the source dataset does not include an explicit treatment assignment, HER2 status was used as a surrogate grouping variable to introduce clinically meaningful heterogeneity in simulated tumor behavior, reflecting well-documented differences in disease response patterns between subgroups [18]. No treatment-effect inference is intended or claimed. A balanced cohort (1:1) was obtained through propensity score matching based on baseline demographic and clinical variables, using logistic regression with nearest-neighbor matching and a caliper of 0.2 standard deviations of the logit of the propensity score [19,20]. Covariate balance was evaluated using standardized mean differences (SMD < 0.1) across key clinical features (Table 1).

The resulting cohort provides a clinically grounded and well-balanced population for simulation while preserving biologically relevant variation in tumor dynamics. Longitudinal tumor trajectories were generated using mechanistic growth models spanning from baseline through nine follow-up visits, yielding 2700 patient-visit observations. This design enables direct and interpretable evaluation of imputation methods while maintaining fidelity to real-world oncology data.

### 2.2. Longitudinal Tumor Size Simulation

Longitudinal tumor size trajectories were generated for each patient across 10 time points (baseline plus nine follow-up visits), resulting in 2700 patient-visit observations. This simulation anchored to the observed baseline tumor size ($S_{0}$), ensuring that subsequent measurements were simulated in a clinically consistent manner.

Tumor dynamics were modeled using biologically motivated growth formulations, incorporating patient-specific clinical and biomarker information to capture heterogeneous progression patterns observed in oncology studies. The primary simulation framework was based on the Gompertz model, which provides a biologically realistic representation of tumor growth, characterized by rapid early expansion followed by deceleration as tumors approach environmental constraints [21].

A unified Gompertz formulation was adopted, where the carrying capacity K represents the maximum tumor burden and the growth rate g reflects intrinsic tumor aggressiveness. Tumor size at time t was defined as $S(t)=K\times exp[\;log(\frac{S_{0}}{K})\times e^{\left(-gt\right)}],$ where $S_{0}$ is the baseline tumor, *K* is the patient- and stage-specific carrying capacity and g is the net growth rate.

To assess the robustness of findings to alternative biological assumptions, a secondary simulation was conducted using the Stein–Fojo bi-exponential model [22].

$S(t)=S_{0}[d\cdot e^{-\lambda t}\;$+ (1 − d) · $e^{\alpha t}]$, where d represents the fraction of drug-sensitive tumor cells, *λ* is the decay rate, and α is the regrowth rate of resistant cells. This formulation enables the generation of more complex, non-linear tumor dynamics commonly observed in oncology settings.

Model parameters in both frameworks were assigned at the patient level using baseline covariates, with covariate-dependent perturbations introduced to reflect differential biological behavior. Lognormal multiplicative noise was added at each post-baseline visit to account for measurement error and biological variability. All simulations were conducted using fixed random seeds to ensure reproducibility.

The purpose of incorporating two distinct simulation models was not to compare biological accuracy, but to evaluate the robustness of downstream analyses under different yet clinically plausible tumor growth dynamics. The Gompertz model served as the primary simulation framework, while the Stein–Fojo model was used for validation. This dual-model design enhances the generalizability of findings by reducing dependence on a single assumed growth mechanism.

### 2.3. RECIST 1.1

A key contribution of this study is the explicit integration of RECIST version 1.1 criteria [2] into the simulation framework, enabling evaluation of missing data methods on clinically meaningful endpoints rather than solely on numerical accuracy. By anchoring the analysis in RECIST-based BOR outcomes, the framework captures the downstream impact of missing data on disease response classification, which is directly relevant to clinical decision-making.

To maintain a controlled and interpretable simulation framework, RECIST was intentionally simplified by representing tumor burden as a single continuous longitudinal measure per patient, serving as a proxy for the sum of target lesion diameters in RECIST 1.1. This approximation captures overall response dynamics while avoiding the complexity of modeling multiple target lesions, non-target lesions, and new lesions. Tumor measurements were generated at clinically relevant follow-up intervals (3, 6, 9, 12, 18, 24, 30, 36, and 48 months) and anchored to each patient’s observed baseline tumor size.

Derived metrics included percent change from baseline and nadir tumor size. Response categories were defined as follows:

- CR: disappearance of lesions;
- PR: ≥30% decrease from baseline;
- SD: between −30% and +20%;
- PD: ≥20% increase from nadir and (≥5 mm absolute increase).

At the patient level, Best Overall Response (BOR) was defined as the best observed response during follow-up (CR > PR > SD > PD) and was derived directly from the simulated tumor trajectories. Progressive disease (PD) required tumor growth exceeding 20% above the nadir at two consecutive assessments. BOR was first determined from the complete trajectories to establish the ground truth and then re-derived after imputation to assess the impact of missing data handling methods.

Simulation parameters were calibrated to produce clinically plausible response distributions consistent with reported breast cancer trials (CR: 5–10%, PR: 55–75%, SD: 20–35%, PD: 10–25%). This calibration ensures that the simulated data reflect realistic treatment response patterns observed in practice [2].

By incorporating RECIST-based BOR assessment into the simulation framework, this study provides a clinically grounded approach for evaluating how missing data handling strategies influence treatment response classification. This helps bridge the gap between methodological performance and clinically meaningful outcomes.

### 2.4. Missing Data Generation

In clinical trials, missing data mechanisms are commonly categorized as Missing Completely at Random (MCAR), Missing At Random (MAR), and Missing Not At Random (MNAR) [5,23]. Missing data were generated under three mechanisms reflecting clinical trial settings:

- MAR (~5%): randomly selected missed visits at data level.
- MNAR-like (~8–15%): progression-related dropout. Progressive disease (PD) was defined as two consecutive assessments showing tumor growth exceeding 20% above the nadir. Once PD was confirmed, all subsequent visits were coded as missing. It is evaluated at data level.
- MAR (~5–10%): non-disease-related dropout due to reasons such as withdrawal of consent or loss to follow-up, resulting in missing observations at subsequent visits at data level.

Total missingness ranged from 20 to 30%, consistent with the documented oncology trials attrition rates [24].

Missing data handling followed an estimand framework recommended in FDA guidance (Table 2) [25]. Progression-related dropout was addressed using a non-responder imputation (NRI) strategy [26], where affected patients were classified as progressive disease (PD), operationalized as tumor size ≥20% above baseline (approximated as 1.2 × $S_{0}$). Missing data assumptions describe why and how data go missing (e.g., MAR or MNAR), whereas estimand strategies determine how those missing observations are handled in the analysis (e.g., direct imputation or NRI).

### 2.5. Model Selection and Imputation Strategy

To evaluate missing data handling in longitudinal tumor trajectories, three imputation methods were employed: Long Short-Term Memory (LSTM) networks [27], MissForest, a non-parametric random forest-based approach [28,29], and Multiple Imputation (MI), implemented by Chained Equations (MICE), a classical statistical imputation method [30]. These methods were selected to represent three widely used approaches to missing data handling: deep learning, machine learning, and classical statistical modeling.

LSTM is a recurrent neural network designed to model temporal dependencies in sequential data [31], making it well suited for longitudinal tumor trajectories. In this study, patient data were represented as sequences and modeled using a two-layer LSTM network (hidden dimension = 64, dropout = 0.25). Input features included tumor size, observation mask, visit time, age, HER2 status, and baseline tumor size. The model was trained using the Adam optimizer (learning rate = 0.003, epoch = 220) with masked mean squared error loss computed only on observed time points. Early stopping (patience = 25) was applied to prevent overfitting, and final imputations were obtained by averaging predictions from 10 forward passes. All analyses were conducted using a fixed random seed (20260324).

MissForest imputes missing values through an iterative random forest procedure without requiring distributional assumptions [29]. Tumor measurements were arranged in wide format and imputed using random forest models (200 trees), incorporating covariates including age, HER2 status, hormone therapy, ER/PR status, and tumor stage. Missing values at each visit were predicted using all other available variables, cycling through visit columns for up to five iterations until convergence. This process was repeated across M = 10 runs, and results were averaged to improve stability.

Multiple Imputation (MI) was implemented using scikit-learn’s IterativeImputer, a MICE-like chained-equations approach based on Bayesian ridge regression [30,32]. Tumor measurements were arranged in wide format and imputed using patient covariates including age, HER2 status, hormone therapy, ER/PR status, and tumor stage. Missing values were iteratively predicted using all other available variables for up to 20 iterations. Posterior sampling was enabled to generate M = 10 independent imputations, and the resulting imputed values were averaged to improve stability.

Extensive hyperparameter tuning was not performed because the objective of the study was to evaluate missing data strategies rather than optimize a specific machine learning model. The same model configuration was applied consistently across all simulation scenarios. All analyses were conducted in Python 3.11 using PyTorch (LSTM), MissForest, and scikit-learn’s IterativeImputer for MI.

Traditional Multiple Imputation methods are commonly applied under the Missing At Random (MAR) assumption [23]. However, this assumption may be violated in oncology studies where missingness is often related to disease progression and treatment discontinuation. Therefore, this study evaluates Multiple Imputation and machine learning-based approaches under clinically realistic missingness scenarios commonly encountered in oncology research.

Therefore, for each imputation method, two missing data handling strategies were implemented: (1) direct imputation, where missing tumor values are estimated based on observed data patterns, and (2) non-responder imputation (NRI). Under NRI, progression-related missingness was treated as non-response, with progressive disease (PD) operationalized as tumor size ≥20% above baseline (approximated as 1.2 × $S_{0}$). Observed values were preserved, and all imputed values were constrained to be ≥0.1 mm to ensure clinical plausibility.

The inclusion of NRI reflects a clinically conservative strategy for handling progression-related missingness and allows comparison between model-based imputation and an estimand-aware approach. Together, this framework enables systematic evaluation of the impact of missing data handling strategies on RECIST-based BOR classification at patient level under clinically realistic missing data scenarios.

### 2.6. Evaluation Metric

Model performance was evaluated using patient-level Best Overall Response (BOR).

Classification performance was assessed using accuracy, Cohen’s kappa [33], F1 score, precision, and recall [34]. These metrics provide a comprehensive evaluation of agreement, classification reliability, and class-level performance across the full cohort (N = 270). In addition, waterfall plots were used to visualize individual tumor changes and overall response patterns.

**Figure 1:**
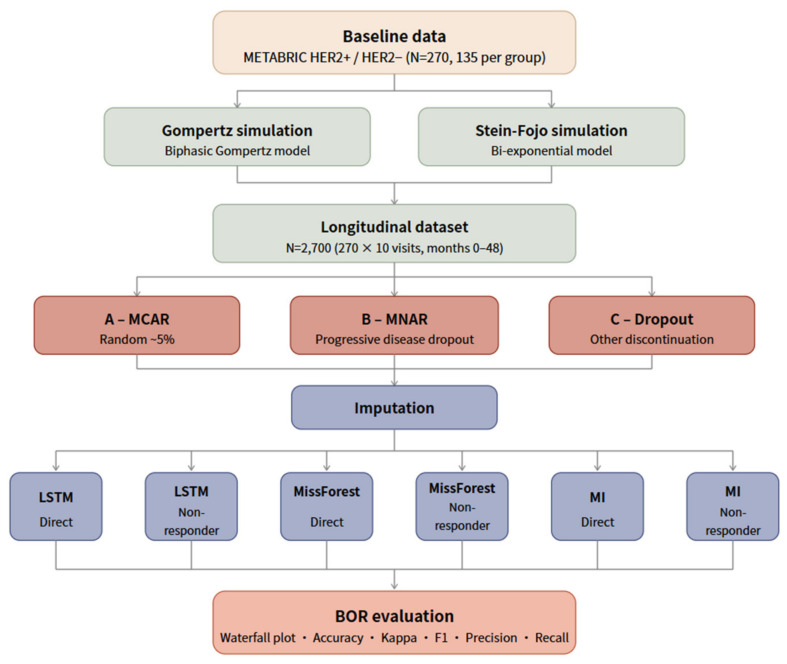
Overview of the simulation and evaluation framework for missing data imputation in longitudinal tumor response analysis using RECIST v1.1–based Best Overall Response (BOR).

Together, these metrics enabled a clinically meaningful assessment of imputation performance by quantifying the accuracy and consistency of RECIST-based response classification under realistic missing data conditions. (Figure 1).

## 3. Results

### 3.1. Gompertz-Simulated Tumor Size Data

**Figure 2:**
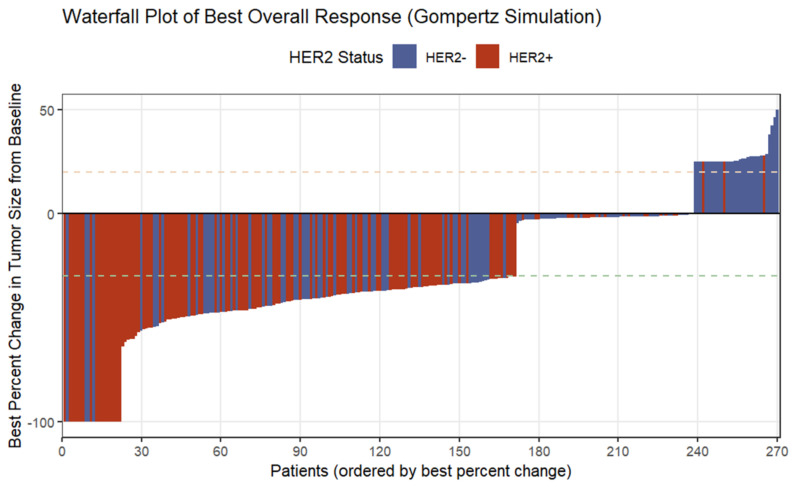
Waterfall plot of best percent change from baseline in tumor size for individual patients from the Gompertz-simulated dataset, stratified by HER2 status. Each bar represents the maximum tumor reduction (or minimum percent change) observed for a patient across all follow-up visits up to 48 months, ordered from greatest reduction to greatest increase. Horizontal dashed lines indicate RECIST thresholds for partial response (−30%) and progressive disease (+20%).

To establish the simulated ground-truth response profile, patient-level tumor responses generated under the Gompertz framework were visualized using a waterfall plot of the best percentage change in tumor size (Figure 2). This representation summarizes the maximum tumor reduction achieved during follow-up and provides a clear overview of response patterns across the HER2 subtypes. This patient-level BOR characterization establishes a clinically grounded reference for evaluating the impact of missing data and imputation methods on response classification.

The simulated responses consisted of 8.1% Complete Response (CR), 55.2% Partial Response (PR), 24.8% Stable Disease (SD), and 11.9% Progressive Disease (PD). These proportions are broadly consistent with RECIST-based response patterns reported in HER2-positive breast cancer trials, where objective response rates typically range from 60 to 80% [35,36]. Overall, the results suggest that the simulation captures clinically meaningful response heterogeneity.

Stratification by HER2 status demonstrates distinct response patterns. HER2+ patients show a higher proportion of tumor reduction (greater representation below −30%), while HER2− patients exhibit relatively more progression (greater proportion above +20%). The SD region is clustered near 0%, reflecting the imposed response structure and ensuring clinically interpretable response boundaries aligned with RECIST criteria.

**Figure 3:**
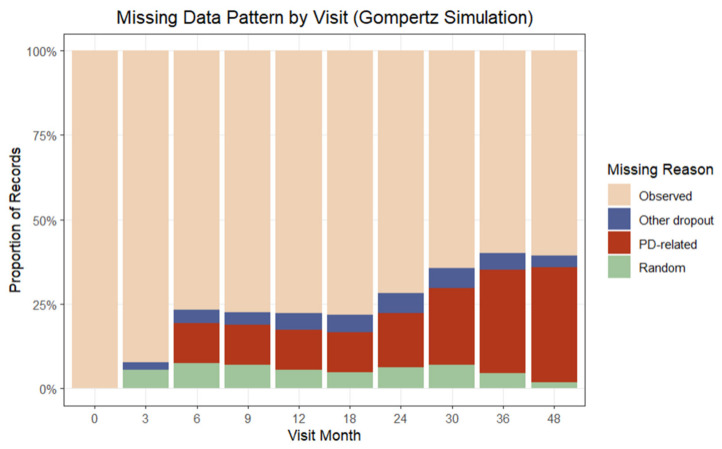
Missing data pattern by visit and reason in Gompertz-simulated tumor trajectories. Stacked bar plot showing the proportion of observed and missing tumor size measurements at each scheduled visit (baseline to 48 months) in the Gompertz-simulated dataset (N = 270 patients).

The simulated missing dataset exhibits an overall missing rate of approximately 25%, comprising ~5% random missingness, 15.1% progression-related dropout, and 4.9% dropout due to other reasons. This level and composition of missingness are consistent with oncology trials, where missing data typically range from 15 to 30% [33]. The visit-level distribution is presented in Figure 3.

These missingness patterns were intentionally designed to reflect clinically realistic mechanisms encountered in oncology studies. Random missingness and non-disease-related dropout approximate Missing At Random (MAR) processes, whereas progression-related dropout represents a Missing Not At Random (MNAR) mechanism driven by underlying disease status. A clear temporal pattern is observed, with progression-related dropout increasing over time and becoming most prominent at later visits (e.g., Month 48). This trend aligns with clinical practice, where patients experiencing disease progression are more likely to discontinue follow-up. Once progression is confirmed (e.g., consecutive PD assessments), subsequent tumor measurements are no longer collected, resulting in monotone missingness in later visits.

Overall, this simulated missing data structure captures clinically plausible dropout behavior and provides a robust framework for evaluating imputation strategies under both MAR and MNAR conditions.

### 3.2. Gompertz-Simulation Patient-Level Response (BOR)

**Figure 4:**
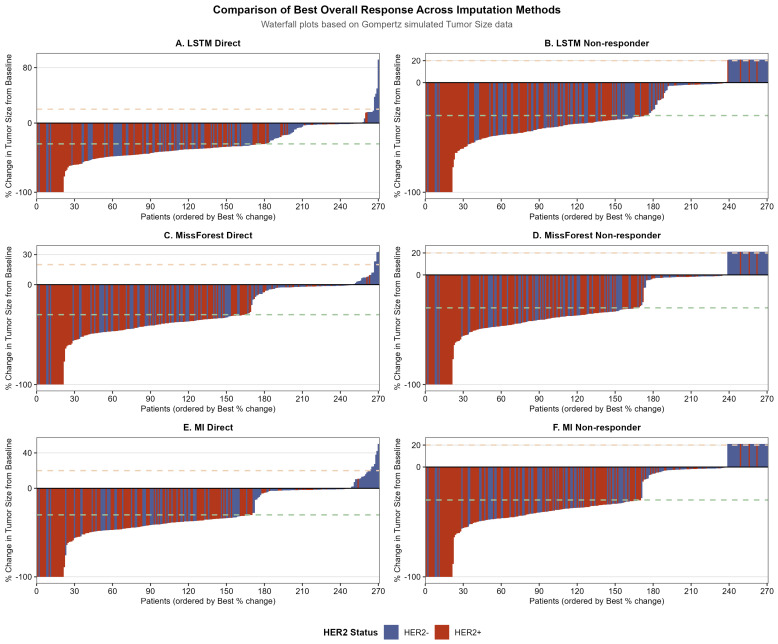
Comparison of Best Overall Response (BOR) across imputation methods using Gompertz-simulated tumor dynamics. Waterfall plots of best percent change in tumor size from baseline for each patient (N = 270), based on Gompertz-simulated longitudinal tumor trajectories. Patients are ordered by their best observed percent change. Siximputation strategies are compared: (**A**) LSTM direct imputation, (**B**) LSTM with non-responder imputation, (**C**) MissForest direct imputation, (**D**) MissForest with non-responder imputation, (**E**) Multiple Imputation direct imputation, and (**F**) Multiple Imputation with non-responder imputation. Horizontal dashed lines indicate RECIST thresholds for PR (−30%) and PD (+20%). Direct imputation assumes missing values are missing at random and are imputed using the model, whereas non-responder imputation assigns progression-consistent values (1.2 × $S_{0}$) for disease-related missingness.

Building on the observed missing data patterns, we next evaluate the performance of patient level BOR in LSTM, MissForest, and MI-based imputation methods under both direct and non-responder imputation (NRI) strategies (Figure 4).

**Figure 5:**
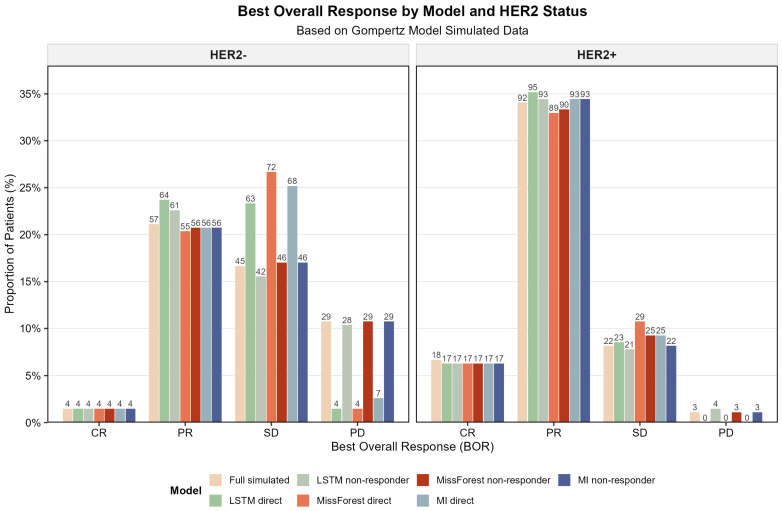
Comparison of Best Overall Response (BOR) across imputation methods stratified by HER2 status. Bar plots showing the distribution of Best Overall Response (BOR) categories, Complete Response (CR), Partial Response (PR), Stable Disease (SD), and Progressive Disease (PD), across imputation methods, stratified by HER2 status (HER2− and HER2+). Results are presented as the proportion of overall population N = 270 and the count for each category.

All threemodels effectively preserved primary response patterns, accurately capturing CR and PR categories (Figure 5). For SD, classification remained relatively stable across methods. NRI produced results that closely aligned with the observed values, whereas direct imputation led to modest and acceptable differences.

The largest differences were observed in the PD category. Under direct imputation, where all missingness is treated as MAR, all three models tended to underestimate PD cases. LSTM generated the most exaggerated tumor size growth trajectories (up to ~80%), whereas MissForest produced more moderate increases (~30%). MI most closely approximated the simulated complete data (~50%). These differences indicate that the imputation methods reconstructed continuous tumor trajectories differently, reflecting distinct modeling assumptions and behaviors.

In contrast, non-responder imputation (NRI) substantially improved the identification of PD cases by explicitly incorporating a clinically informed progression assumption for progression-related dropout. This was identical to the simulated complete dataset (32 patients in the simulated data and across the LSTM, MissForest, and MI models under NRI). Although NRI does not attempt to reconstruct the underlying tumor trajectory, it improves BOR classification by assigning a conservative non-response status, resulting in less variability in the imputed trajectories.

Together, these findings highlight a key trade-off: direct imputation better preserves continuous tumor dynamics but may underestimate progression events, whereas non-responder imputation improves clinically relevant classification of PD while simplifying underlying trajectories. Consequently, the optimal missing data strategy depends on the study objective and endpoint of interest. This distinction is particularly important in clinical trials, where different imputation approaches may lead to different conclusions for continuous tumor-based endpoints versus categorical response outcomes.

To quantitatively assess agreement with the reference BOR, we evaluated accuracy, Cohen’s kappa, and macro-averaged precision, recall, and F1 score across methods. Direct imputation achieved relatively high accuracy (0.84 for LSTM, 0.87 for MissForest, and 0.89 for MI), driven largely by strong performance in dominant categories (PR and CR). However, agreement metrics were lower, with kappa values between 0.73 and 0.82, and macro F1 scores of 0.72–0.79, reflecting reduced performance in less frequent but clinically critical categories, particularly PD (Table 3).

Non-responder imputation markedly improved performance across all metrics. Accuracy increased to 0.94 (LSTM), 0.98 (MissForest), and 0.99 (MI) with kappa values of 0.9, 0.97 and 0.99, indicating near-perfect agreement. Macro F1 scores also improved substantially (0.95–0.99), demonstrating more balanced classification across all response categories. These gains were primarily driven by improved identification of PD events.

These results highlight that direct imputation preserves continuous tumor response trajectories better but may underestimate progression events and reduce agreement in RECIST-based classifications. In contrast, non-responder imputation, which is aligned with estimand-aware clinical trial practice, improves progression detection and overall classification agreement, although it simplifies the underlying tumor growth trajectories.

### 3.3. Stein–Fojo Simulated Tumor Size Data

**Figure 6:**
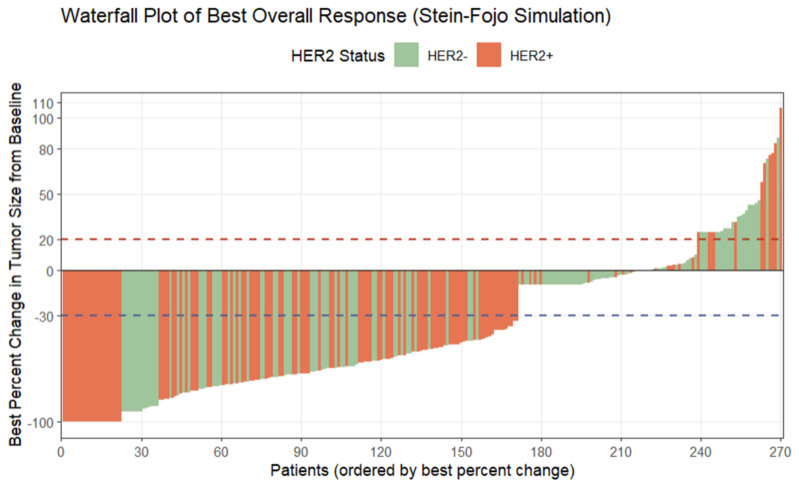
Waterfall plot of best percent change from baseline in tumor size for individual patients from the Stein–Fojo simulated dataset, stratified by HER2 status. Each bar represents the maximum tumor reduction (or minimum percent change) observed for a patient across all follow-up visits up to 48 months, ordered from greatest reduction to greatest increase. Horizontal dashed lines indicate RECIST thresholds for partial response (−30%) and progressive disease (+20%).

To evaluate the robustness of our findings to the underlying tumor growth assumptions, we conducted a validation analysis using the Stein–Fojo simulation framework. Compared to the Gompertz model, the Stein–Fojo simulation exhibits more aggressive tumor growth, with maximum percent change exceeding 100%, and a smoother transition around the stable disease region near 0% change (Figure 6).

**Figure 7:**
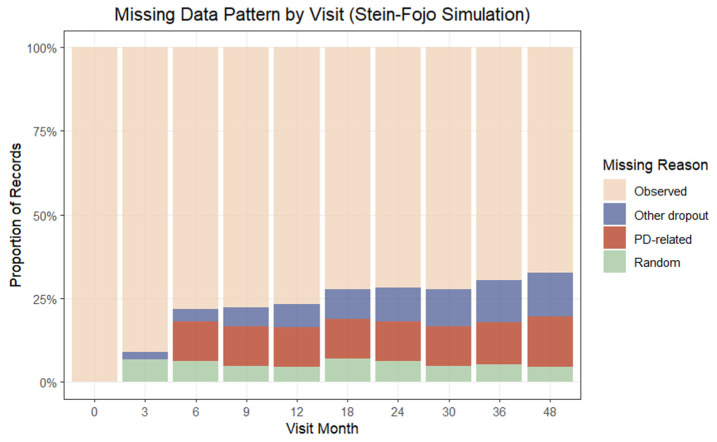
Missing data pattern by visit and reason in Stein–Fojo simulated tumor trajectories. Stacked bar plot showing the proportion of observed and missing tumor size measurements at each scheduled visit (baseline to 48 months) in the Stein–Fojo simulated dataset (N = 270 patients).

To ensure comparability, the BOR distribution of Stein–Fojo was constrained to match that of the Gompertz framework (CR: 8.1%, PR: 55.2%, SD: 24.8%, PD: 11.9%), while maintaining a similar missing data structure (5% random missingness, 9.9% PD-related dropout, and 7.4% other dropout; total 22.3%) (Figure 7). This preserves clinically realistic missing data mechanisms while allowing evaluation under an alternative tumor growth model.

### 3.4. Stein–Fojo Patient-Level Response (BOR)

**Figure 8:**
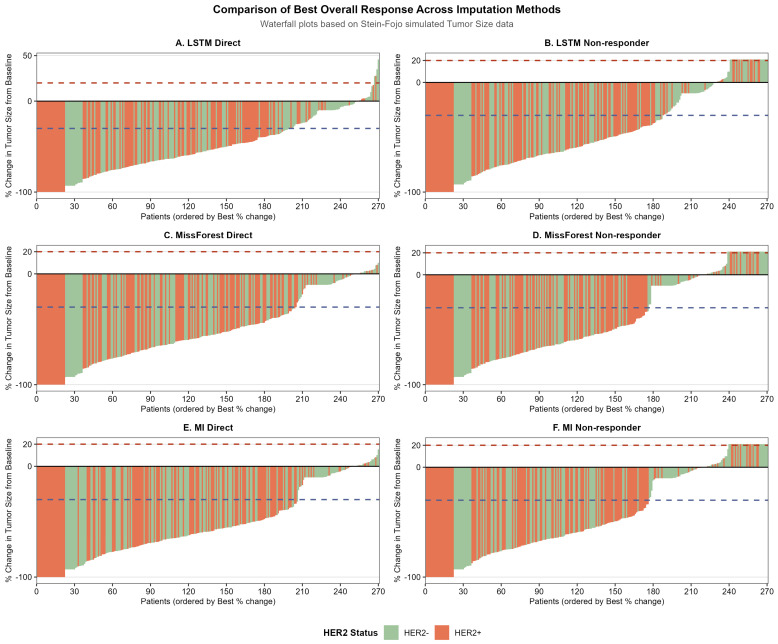
Comparison of Best Overall Response (BOR) across imputation methods using Stein–Fojo simulated tumor dynamics. Waterfall plots of best percent change in tumor size from baseline for each patient (N = 270). Patients are ordered by their best observed percent change. Six imputation strategies are compared: (**A**) LSTM direct imputation, (**B**) LSTM with non-responder imputation, (**C**) MissForest direct imputation, (**D**) MissForest with non-responder imputation, (**E**) MI direct imputation, and (**F**) MI with non-responder imputation. Horizontal dashed lines indicate RECIST thresholds for PR (−30%) and PD (+20%). Direct imputation assumes missing values are missing at random and are imputed using the model, whereas non-responder imputation assigns progression-consistent values for disease-related missingness.

**Figure 9:**
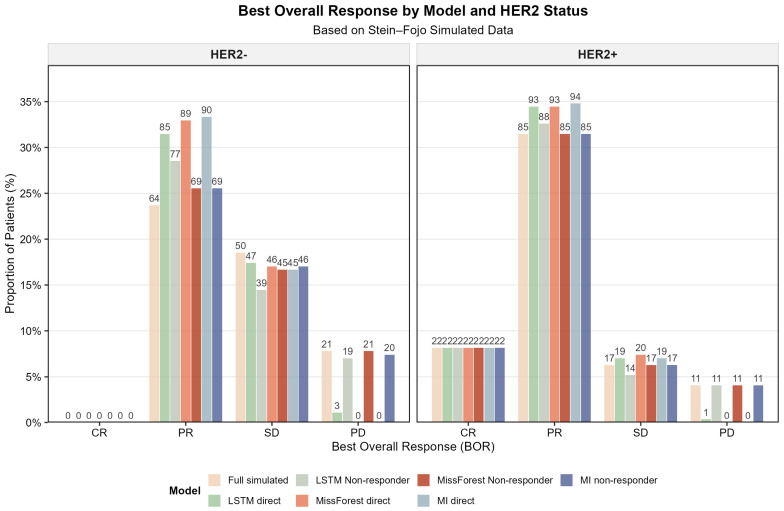
Comparison of Best Overall Response (BOR) across imputation methods stratified by HER2 status. Bar plots showing the distribution of Best Overall Response (BOR) categories, Complete Response (CR), Partial Response (PR), Stable Disease (SD), and Progressive Disease (PD), across imputation methods, stratified by HER2 status. Results are presented as the proportion of overall population N = 270 and the count for each category.

The resulting response patterns are consistent with the Gompertz-based analysis. HER2+ patients demonstrate a more favorable response profile, with greater tumor shrinkage and all complete responses occurring within the HER2+ group (Figure 8). Across three imputation algorithms, LSTM, MissForest, and MI, the dominant BOR categories, particularly CR, PR and SD, were consistently reproduced, with differences primarily observed in the identification of progressive disease (PD) (Figure 9).

Direct imputation continues to underrepresent PD events, with all direct imputation failing to recover PD cases regardless of the models. In contrast, non-responder imputation (NRI) substantially improves classification performance again. Accuracy and Cohen’s kappa increase from 0.82–0.84 and 0.69–0.72 under direct imputation to 0.91–0.96 and 0.86–0.94 under non-responder imputation, with corresponding improvements in F1 score and recall (Table 4), indicating enhanced detection of clinically meaningful progression events at categorical level.

## 4. Discussion

This study provides a clinically grounded evaluation of missing data handling in oncology using a METABRIC-derived simulation framework and RECIST-based Best Overall Response (BOR) as the primary endpoint. Consistent findings across the Gompertz and Stein–Fojo models support the robustness of the results under different tumor growth assumptions.

The contribution of this study is three-fold. First, this study shifts the focus from merely reconstructing missing tumor size measurements to evaluating how imputation choices influence clinically meaningful outcomes. By linking tumor size with RECIST-based BOR classification, we show that imputation methods that achieve accurate categorical treatment response classifications may not necessarily provide accurate reconstruction of continuous tumor measurements, highlighting the critical role of endpoint selection in the evaluation of missing data methods.

Second, this study demonstrates that clinical estimand specification can be more influential than algorithmic complexity in handling missing data. Non-responder imputation improved BOR classification by approximately 10 percentage points across all evaluated methods and both simulation frameworks. Furthermore, the finding indicates that NRI is not simply a conservative regulatory convention; it was consistently superior across scenarios, suggesting that regulatory practice and empirical performance are aligned.

Third, this study highlights the importance of realistic missingness mechanisms in evaluating imputation methods. Under MAR, most methods performed reasonably well regardless of estimand choice. However, under progression-driven MNAR, the more clinically realistic setting in oncology trials, only estimand-aligned approaches such as NRI consistently captured disease progression, whereas direct imputation tended to underestimate progression events. These findings underscore the need for evaluation frameworks that incorporate realistic missingness assumptions to support clinically reliable conclusions.

Taken together, these findings suggest that successful handling of missing data in longitudinal oncology studies depends not only on the imputation method itself, but also on the choice of clinically meaningful endpoints and estimand strategies appropriate for the underlying missing data mechanism. When treatment response was evaluated using the patient-level RECIST-based BOR endpoint, estimand-aware approaches such as non-responder imputation (NRI) under progression-related missingness consistently improved classification performance across LSTM, MissForest, and Multiple Imputation. Overall, the findings highlight the importance of matching endpoint definition and missing data handling strategies to the clinical context when evaluating imputation methods in longitudinal tumor size research.

Several limitations should be considered. First, this study is based on simulated longitudinal data, with tumor burden represented as a single continuous measure rather than the sum of multiple target lesions used in clinical practice. Consequently, the framework does not fully capture the complexity of RECIST assessment, including multiple lesion trajectories and new lesion emergence. However, the simulation framework was anchored to the METABRIC cohort to preserve clinically relevant heterogeneity. Moreover, the consistency of findings across the Gompertz and Stein–Fojo models supports the robustness of the conclusions. Future work could extend this framework using fully observed longitudinal datasets and more comprehensive representations of tumor burden. In addition, integrating longitudinal tumor dynamics with survival outcomes through joint modeling may provide a more comprehensive framework for evaluating missing data strategies and their impact on both response and time-to-event endpoints.

Second, the primary endpoint was patient-level Best Overall Response (BOR), a categorical outcome. The strong performance of non-responder imputation (NRI) reflects its ability to improve progressive disease classification rather than reconstruct the underlying tumor trajectory. As shown in the waterfall plots, NRI preserves clinically relevant response categories but does not fully recover continuous tumor dynamics. Consequently, these findings may not generalize to continuous tumor-based endpoints or group-level treatment effect analyses, where accurate trajectory reconstruction may be more important. Future work should evaluate whether more sophisticated longitudinal models and optimized machine learning approaches can better recover continuous tumor trajectories while maintaining clinically meaningful response classification.

Third, the non-responder imputation strategy applies a simplified rule by assigning progression-related missing values based on a fixed threshold (≥1.2 × baseline tumor size). While this approach aligns with estimand-based strategies commonly used in clinical trials and improved BOR classification in the present study, it may not fully capture the diversity of progression patterns observed in practice. More fundamentally, outcomes following progression-related dropout are inherently unobserved, meaning that any approach for handling Missing Not At Random (MNAR) data must rely on assumptions that cannot be empirically verified. This limitation also highlights the value of simulation-based evaluation: unlike real clinical datasets, where the true post-dropout trajectory is unknown, simulation provides a controlled setting in which the impact of different missing data assumptions and imputation strategies on treatment response classification can be systematically assessed.

Despite these limitations, this study provides a clinically grounded framework for evaluating missing data methods using RECIST-based BOR classification within a METABRIC-derived simulation setting. By jointly examining endpoint definition and estimand-aware strategy with realistic missingness mechanisms, this study demonstrates how these factors influence response classification. The findings underscore the importance of considering both components when evaluating missing data methods in oncology research.

## 5. Conclusions

This study presents a clinically grounded evaluation of machine learning-based imputation methods for longitudinal oncology data using RECIST-based Best Overall Response (BOR) as the primary endpoint. Complete tumor trajectories were generated from METABRIC-derived baseline characteristics using both Gompertz and Stein–Fojo growth models, with missingness introduced under clinically relevant MAR, progression-driven MNAR, and monotone dropout scenarios. This simulation framework preserves the underlying ground truth and enables objective evaluation of missing data methods under controlled conditions.

The contributions of this study are threefold. First, it shifts the evaluation of imputation methods from numerical reconstruction of tumor measurements to clinically meaningful RECIST-based BOR classification. Second, it demonstrates the importance of estimand-aware handling of progression-related missing data, showing that clinically informed assumptions can substantially influence patient-level treatment response assessment. Third, it highlights the critical role of realistic missingness mechanisms, particularly progression-driven MNAR scenarios, in the evaluation of missing data methods for oncology studies.

Across both simulation frameworks, non-responder imputation (NRI) consistently improved BOR classification regardless of the underlying imputation method. These findings suggest that successful handling of missing data depends not only on the imputation algorithm itself, but also on the clinical endpoint and the corresponding estimand strategy aligned with the underlying missing data assumptions. In the METABRIC-derived simulations, clinically informed handling of progression-related missingness had a greater influence on BOR classification than differences among the evaluated imputation methods. Overall, the results suggest that endpoint definition and estimand-aware missing data handling may be more influential than the choice of imputation algorithm in longitudinal oncology studies.

## Figures and Tables

**Table 1:** Baseline characteristics of the simulated cohort.

Category	Variable	HER2 Negative (*n* = 135)	HER2 Positive (*n* = 135)
Demographics	Age at Diagnosis (years), Mean ± SD	56.68 ± 13.64	55.70 ± 13.39
	Menopausal state (post-menopausal, %)	28.90%	36.30%
Biomarkers	ER positive (%)	63.00%	44.40%
	PR positive (%)	25.90%	23.00%
Tumor Burden	Baseline tumor size (mm), Mean ± SD	28.00 ± 15.25	28.73 ± 17.23
	Tumor stage II (%)	57.80%	56.30%
	Positive lymph nodes, Mean	3.13	3.21
	Histologic grade 3 (%)	84.40%	83.00%
Prior Line Treatment	Received chemotherapy (%)	31.10%	43.00%
	Received hormone therapy (%)	65.20%	51.10%

*ER = estrogen receptor; PR = progesterone receptor; HER2 = human epidermal growth factor receptor 2; SD = standard deviation.*

**Table 2:** Estimand Strategy.

Estimand Attribute	Description
**Population**	The 270-patient clinically anchored matched cohort (*n* = 135 HER2-positive, *n* = 135 HER2-negative).
**Treatment**	HER2 subgroup comparison used as the exposure grouping.
**Endpoint**	Best Overall Response (BOR) or responder status derived from simulated longitudinal tumor measurements using RECIST-based criteria.
**Intercurrent Event**	Progression-related dropout handled using non-responder imputation (NRI), with such patients classified as progressive disease (PD). PD is conservatively set as tumor size ≥20% above baseline (≈1.2 × S_0_ baseline).
**Summary Measure**	Proportion of patients’ BOR in each response category (CR, PR, SD, PD) by HER2 group.

*BOR = Best Overall Response; CR = complete response; HER2 = human epidermal growth factor receptor 2; NRI = non-responder imputation; PD = progressive disease; PR = partial response; RECIST = Response Evaluation Criteria in Solid Tumors; SD = stable disease; S_0_ = baseline tumor size.*

**Table 3:** Performance evaluation of direct and non-responder missing data strategies across classification models. Performance evaluation of imputation methods for multi-class response classification (CR, PR, SD, PD). Metrics were calculated by comparing model-predicted responses with the reference classification derived from the fully simulated dataset. Non-responder imputation strategies are included.

Model	Accuracy	Kappa	F1 Score	Precision	Recall
LSTM—Direct	0.841	0.729	0.723	0.895	0.727
LSTM—Non-responder	0.941	0.902	0.947	0.956	0.939
MissForest—Direct	0.870	0.786	0.743	0.916	0.759
MissForest— Non-responder	0.981	0.970	0.983	0.984	0.981
MI—Direct	0.893	0.821	0.787	0.924	0.786
MI—Non-responder	0.992	0.988	0.991	0.995	0.987

**Table 4:** Performance evaluation of direct and non-responder missing data strategies across classification models. Performance evaluation of imputation methods for multi-class response classification (CR, PR, SD, PD). All metrics were calculated by comparing model-predicted responses with the reference classification derived from the fully simulated dataset. Non-responder imputation strategy is tumor size = 1.2 × $S_{0}$.

Model	Accuracy	Kappa	F1 Score	Precision	Recall
LSTM—Direct	0.822	0.693	0.716	0.896	0.722
LSTM—Non-responder	0.914	0.859	0.916	0.955	0.889
MissForest—Direct	0.841	0.721	0.934	0.901	0.730
MissForest— Non-responder	0.963	0.939	0.961	0.981	0.945
MI—Direct	0.837	0.714	0.932	0.903	0.727
MI—Non-responder	0.956	0.928	0.951	0.973	0.935

## Data Availability

The METABRIC breast cancer dataset used in this study is publicly available via cBioPortal at https://www.cbioportal.org/study/clinicalData?id=brca_metabric (accessed on 8 January 2026). The simulation datasets generated during this study are available from the corresponding author upon reasonable request.

## References

[1] Delgado A., Guddati A.K. (2021). Clinical endpoints in oncology: A primer. Am. J. Cancer Res..

[2] Eisenhauer E.A., Therasse P., Bogaerts J., Schwartz L.H., Sargent D., Ford R., Verweij J. (2009). New response evaluation criteria in solid tumours: Revised RECIST guideline (version 1.1). Eur. J. Cancer.

[3] Hopkins A.M., Kichenadasse G., McKinnon R.A., Rowland A., Sorich M.J. (2019). Baseline tumor size and survival outcomes in lung cancer patients treated with immune checkpoint inhibitors. Seminars in Oncology.

[4] Michaelson J.S., Silverstein M., Wyatt J., Weber G., Moore R., Halpern E., Kopans D.B., Hughes K. (2002). Predicting the survival of patients with breast carcinoma using tumor size. Cancer.

[5] Rubin D.B. (1976). Inference and missing data. Biometrika.

[6] Yang D.X., Khera R., Miccio J.A., Jairam V., Chang E., Yu J.B., Aneja S. (2021). Prevalence of missing data in the national cancer database and association with overall survival. JAMA Netw. Open.

[7] Mandrekar S.J., An M.W., Meyers J., Grothey A., Bogaerts J., Sargent D.J. (2014). Evaluation of alternate categorical tumor metrics and cut points for response categorization using the RECIST 1.1 data warehouse. J. Clin. Oncol..

[8] Jain R.K., Lee J.J., Ng C., Hong D., Gong J., Naing A., Wheler J., Kurzrock R. (2012). Change in tumor size by RECIST correlates linearly with overall survival in phase I oncology studies. J. Clin. Oncol..

[9] Claret L., Girard P., Hoff P.M., Van Cutsem E., Zuideveld K.P., Jorga K., Fagerberg J., Bruno R. (2009). Model-based prediction of phase III overall survival in colorectal cancer on the basis of phase II tumor dynamics. J. Clin. Oncol..

[10] Lim H.-S., Sun W., Parivar K., Wang D. (2019). Predicting overall survival and progression-free survival using tumor dynamics in advanced breast cancer patients. AAPS J..

[11] Jerez J.M., Molina I., García-Laencina P.J., Alba E., Ribelles N., Martín M., Franco L. (2010). Missing data imputation using statistical and machine learning methods in a real breast cancer problem. Artif. Intell. Med..

[12] Joel L.O., Doorsamy W., Paul B.S. (2025). A comparative study of imputation techniques for missing values in healthcare diagnostic datasets. Int. J. Data Sci. Anal..

[13] Twala B., Jones M.C., Hand D.J. (2020). Accuracy of random-forest-based imputation of missing data in the presence of non-normality, non-linearity, and interaction. BMC Med. Res. Methodol..

[14] Che Z., Purushotham S., Cho K., Sontag D., Liu Y. (2018). Recurrent neural networks for multivariate time series with missing values. Sci. Rep..

[15] Kazijevs M., Samad M.D. (2023). Deep imputation of missing values in time series health data: A review with benchmarking. J. Biomed. Inform..

[16] Ren W., Liu Z., Wu Y., Zhang Z., Hong S., Liu H., Missing Data in Electronic health Records (MINDER) Group (2024). Moving Beyond Medical Statistics: A Systematic Review on Missing Data Handling in Electronic Health Records. Health Data Sci..

[17] Curtis C., Shah S.P., Chin S.F., Turashvili G., Rueda O.M., Dunning M.J., Aparicio S. (2012). The genomic and transcriptomic architecture of 2,000 breast tumours reveals novel subgroups. Nature.

[18] Tan F., Zhou J.-G., Li S., Long B., Bellur S., Zhou Y., Newman M. (2026). A unified framework for survival prediction: Combining machine learning feature selection with traditional survival analysis in heart failure and METABRIC breast cancer. Diagnostics.

[19] Haukoos J.S., Lewis R.J. (2015). The propensity score. JAMA.

[20] Kernan W.N., Viscoli C.M., Makuch R.W., Brass L.M., Horwitz R.I. (1999). Stratified randomization for clinical trials. J. Clin. Epidemiol..

[21] Tjørve K.M., Tjørve E. (2017). The use of Gompertz models in growth analyses, and new Gompertz-model approach: An addition to the unified-Richards family. PLoS ONE.

[22] Stein W.D., Figg W.D., Dahut W., Stein A.D., Hoshen M.B., Price D., Bates S.E., Fojo T. (2008). Tumor growth rates derived from data for patients in a clinical trial correlate strongly with patient survival: A novel strategy for evaluation of clinical trial data. Oncologist.

[23] National Research Council (2010). The Prevention and Treatment of Missing Data in Clinical Trials.

[24] Hui D., Glitza I., Chisholm G., Yennu S., Bruera E. (2013). Attrition rates, reasons, and predictive factors in supportive care and palliative oncology clinical trials. Cancer.

[25] Akacha M., Bretz F., Ruberg S. (2017). Estimands in clinical trials: Broadening the perspective. Stat. Med..

[26] European Medicines Agency (2010). Guideline on Missing Data in Confirmatory Clinical Trials (EMA/CPMP/EWP/1776/99 Rev.1). https://www.ema.europa.eu/en/documents/scientific-guideline/guideline-missing-data-confirmatory-clinical-trials_en.pdf.

[27] Hochreiter S., Schmidhuber J. (1997). Long short-term memory. Neural Comput..

[28] Breiman L. (2001). Random forests. Mach. Learn..

[29] Stekhoven D.J., Bühlmann P. (2012). MissForest: Non-parametric missing value imputation for mixed-type data. Bioinformatics.

[30] Rubin D. (1987). Multiple Imputation for Nonresponse in Surveys.

[31] Sterne J.A.C., White I.R., Carlin J.B., Spratt M., Royston P., Kenward M.G., Wood A.M., Carpenter J.R. (2009). Multiple imputation for missing data in epidemiological and clinical research: Potential and pitfalls. BMJ.

[32] Pedregosa F., Varoquaux G., Gramfort A., Michel V., Thirion B., Grisel O., Blondel M., Prettenhofer P., Weiss R., Dubourg V. (2011). Scikit-learn: Machine learning in Python. J. Mach. Learn. Res..

[33] Cohen J. (1960). A coefficient of agreement for nominal scales. Educ. Psychol. Meas..

[34] Sokolova M., Lapalme G. (2009). A systematic analysis of performance measures for classification tasks. Inf. Process. Manag..

[35] Baselga J., Cortés J., Kim S.B., Im S.A., Hegg R., Im Y.H., Swain S.M. (2012). Pertuzumab plus trastuzumab plus docetaxel for metastatic breast cancer. N. Engl. J. Med..

[36] Rugo H.S., Barve A., Waller C.F., Hernandez-Bronchud M., Herson J., Yuan J., Pennella E. (2017). Effect of a proposed trastuzumab biosimilar compared with trastuzumab on overall response rate in patients with ERBB2 (HER2)–positive metastatic breast cancer: A randomized clinical trial. JAMA.

